# In Situ Observation of the Thermal Behavior of Graphene on Insulating and Metal Substrates

**DOI:** 10.3390/nano15070557

**Published:** 2025-04-05

**Authors:** Mikihiro Kato, Xinwei Zhao

**Affiliations:** Department of Physics, Tokyo University of Science, 1–3 Kagurazaka, Shinjuku 162-8601, Tokyo, Japan; xwzhao@rs.tus.ac.jp

**Keywords:** in situ observation, graphene, Raman spectra

## Abstract

In general, graphene is known to be thermally stable. In this study, we analyzed the Raman spectra of graphene prepared on copper (Cu) and nickel (Ni) by chemical vapor deposition (CVD) as well as monolayer and multilayer graphene transferred onto SiO_2_ under vacuum heating. We observed a shift in the position of the graphene G peak due to temperature changes for all substrates. For graphene on insulating substrates, the peak position returned to its original position after heating when the substrate returned to room temperature, indicating the thermal and chemical stability of graphene. In contrast, the Raman spectra of graphene on Cu and Ni, which have different carbon solubilities, showed significant shifts and broadening of the G peak as the temperature increased. We also utilized optical microscopy to observe morphological changes during heating, which complemented the Raman spectroscopy analysis. The optical microscopy images obtained in the previous study revealed morphological changes on the graphene surface that correlate with the shifts observed in the Raman spectra, especially in graphene on metal substrates. These combined findings from Raman spectroscopy and optical microscopy could provide insights for optimizing graphene growth processes. In addition, knowledge of the thermal behavior of graphene on insulating substrates could be useful for device construction.

## 1. Introduction

Nearly 20 years after the Nobel Prize was awarded for graphene’s properties, much research is still being conducted on this remarkable material.

Graphene is generally produced by thermal CVD using catalytic metals. The carbon solubility in the catalytic metals and the growth mechanism are different [1]. In recent years, research on graphene growth using low-temperature CVD (LT-CVD) has been progressing, and the adsorption and decomposition mechanism of liquid aromatic hydrocarbon precursors on the Cu (111) surface has been theoretically analyzed [2]. On the other hand, heterostructures of transition metal dichalcogenides (TMDCs) and graphene have also attracted attention. For example, Bianco et al. reported the growth of uniform, high-quality WS_2_ films by directly epitaxially CVD-synthesizing tungsten disulfide (WS_2_) on epitaxially and CVD-grown graphene [3]. These studies deepen our understanding of graphene growth using the CVD method and contribute to the design of optimal growth conditions.

Raman spectroscopy and optical microscopy are widely used as effective tools for the evaluation of carbon materials. Raman spectra can provide information on chemical bonding, crystallinity, and crystal lattice distortions. Moreover, Raman spectroscopy allows for in situ observation in a non-destructive and non-contact manner. For graphene evaluation, a sharp peak (G-band) can be observed around 1580 cm^−1^. As the number of layers increases, the peak at around 2700 cm^−1^ (2D band) becomes broader in proportion to the number of layers. Additionally, the normally inactive peaks at 1360 cm^−1^ (D-band) and 1620 cm^−1^ (D’-band) appear when the structure is disordered or defective. Such Raman spectra are used to assess the crystallinity and number of layers of graphene [4,5,6,7,8]. On the other hand, optical microscopy is employed to observe graphene by utilizing interference and reflection with the substrate. Notably, when observing graphene on SiO_2_, it can be clearly visualized due to the interference between the oxide film and the graphene [9,10,11]. However, Raman measurements are typically conducted for graphene on insulating substrates and observed at room temperature in air, which only provides partial information about the material. Furthermore, there are limited studies that investigate the effects of varying the measurement environment. In this study, we investigated the potential changes in the graphene characteristic peaks when graphene synthesized by CVD and transferred onto SiO_2_, as well as single-layer and multilayer graphene and graphene on metal foils (Cu and Ni), were heated and cooled in a vacuum.

The thermal behavior of graphene on insulating substrates was evaluated using in situ optical microscopy and Raman spectroscopy, and it was confirmed that graphene is chemically and structurally stable at high temperatures. This suggests the possibility of device applications in high-temperature environments. In addition, by demonstrating that graphene is stable on SiO_2_, it is possible to relatively evaluate the dissolution and alteration of graphene on metal substrates by in situ observation.

## 2. Experimental

### 2.1. CVD-Grown Graphene Films

Polycrystalline Ni foil (>99% purity, The Nilaco Corporation, Chuo, Japan) was used as the starting sample. The surface was coated with a thin layer of carbon, as disordered few-layer graphene, grown by thermal CVD. The carbon deposition was carried out with ethanol vapor, generated by bubbling Ar gas through liquid ethanol, at 870 °C for 30 min under a total pressure of 5 Torr. Similarly, graphene films were prepared using polycrystalline Cu foil (>99.99% purity, The Nilaco Corporation, Chuo, Japan) as the substrate. The deposition was performed at 950 °C for 10 min by thermal CVD with ethanol vapor generated as described above, under a total pressure of 5 Torr.

For graphene on SiO_2_, a commercial sample (Graphene Platform Corp., Shibuya, Japan) was used in the experiments.

### 2.2. Characterization Techniques

**Figure 1:**
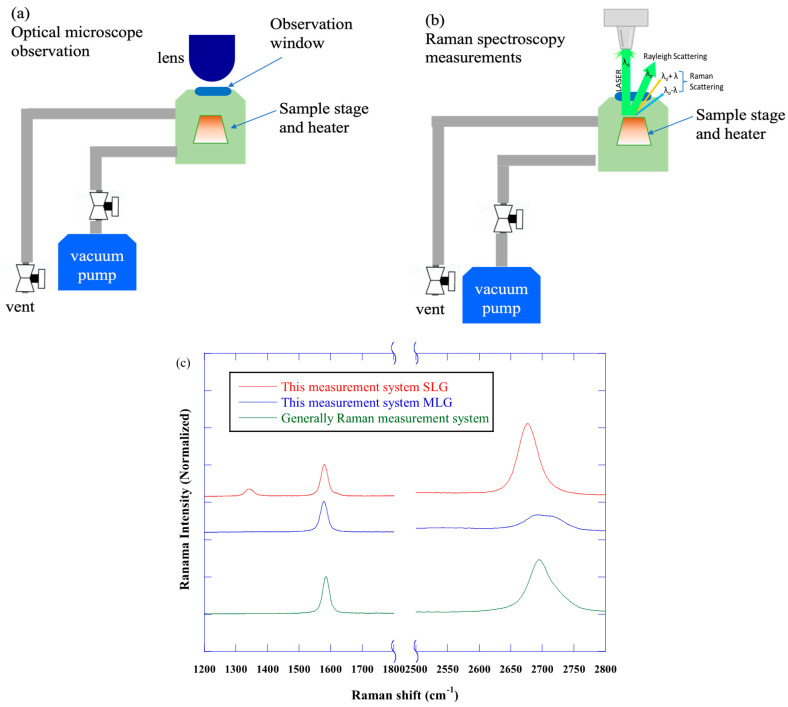
Schematic diagram of the measurement system. (**a**) shows the optical image observation mode and (**b**) the Raman measurement mode. The sample is heated via a ceramic sample stage. The temperature of the sample stage is measured using thermocouples (Armel–Chromel thermocouples). (**c**) shows the Raman spectra of single-layer and multi-layer graphene at room temperature measured with our system and with a conventional Raman spectrometer.

Figure 1 shows the observation system. The optical microscope (KH-7700, Hirox, Suginami, Japan) and Raman spectroscopy measurements (i-Raman EX, B&W TEK, Plainsboro, NJ, US) were used for observation and measurement. The optical microscope’s objective lens had a magnification range from ×140 to ×1400, and the light source was a metal halide lamp. The heating stage for the in situ observation was set in a vacuum chamber (S.T. Japan Co., Ltd., Chuo, Japan) with a quartz observation window. The temperature range was from room temperature (RT) to 900 °C. The samples were heated by a heater installed in a small chamber. The observation conditions were in vacuum (~10^−6^ Torr) and the heating temperature was from RT to 900 °C. When 900 °C was reached, the temperature was maintained at 900 °C for about 5 min and then cooled down to RT by natural cooling. The Raman spectra were recorded at every 100 °C under the above conditions to monitor changes in the graphene-specific spectral features. The Raman spectra were also measured every 100 °C during the cooling process from 900 °C to RT to see if there was any change in the Raman spectra during heating and cooling. Raman spectra were measured by irradiating a laser beam through the observation window at the top of the chamber. The wavelength of the laser beam used for Raman spectroscopy was 532 nm. The maximum laser power was 430 mW. The data of a Raman spectrum were obtained by irradiating the sample 10 times for 3 s.

As shown in Figure 1, Raman measurement was performed through the observation window of a small chamber, so an objective lens (Olympus corporation, Shinjuku, Japan) with a working distance of 25 mm was used. The optical system was different for optical image observation and Raman measurement. As shown in Figure 1c, we confirmed that the measurements were not particularly unusual.

## 3. Results and Discussion

### 3.1. Raman Spectra of Transferred Graphene on SiO_2_ Substrate

**Figure 2:**
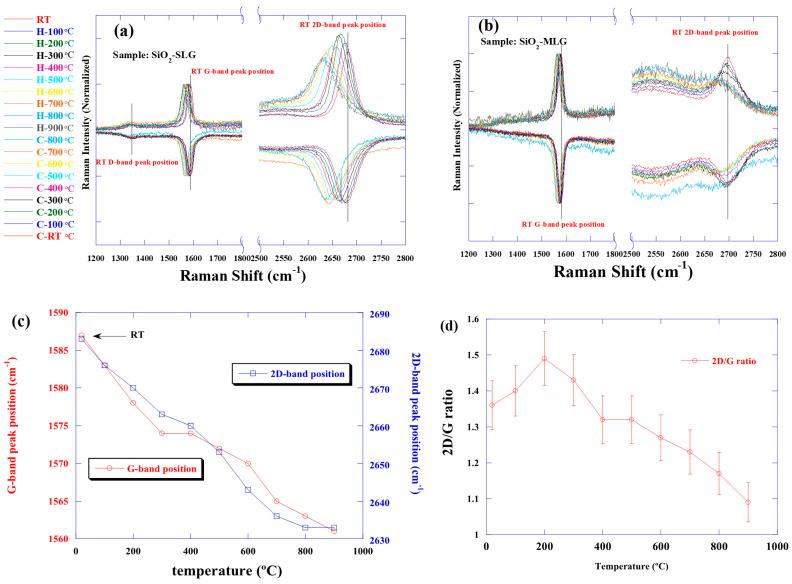
(**a**) (left figure) single layer graphene and (**b**) (right figure) multilayer graphene on SiO_2_ substrate. The relationship between the spectrum during heating and cooling is shown. The results for cooling are inverted to confirm the peak position at each temperature. (**c**) is the relationship between temperature and peak position of single layer graphene on SiO_2_ substrate. The peak position of the maximum value for each temperature is noted. (**d**) is the relationship between temperature and peak intensity ratio. The 2D/D ratio was found to decrease with increasing temperature.

The Raman spectra of graphene transferred onto an insulating substrate are presented in Figure 2a,b. The G-band and 2D band peaks are at the same position for both monolayer and multilayer graphene at the same temperature, after heating and cooling, and at RT before and after heating. In general, the peak shifts to a lower wavelength when the Raman spectra are measured at elevated temperature. The change in the phonon frequency with temperature manifests the anharmonic terms in the lattice potential energy, which is determined by the anharmonic potential constants, the phonon occupation number, and the thermal expansion of the crystal [12].

The relationship between temperature and peak position is shown in Figure 2c. Heating from RT to 900 °C resulted in a shift of 26 cm^−1^ in the G-band and 50 cm^−1^ in the 2D band, and the slopes of the G- and 2D band versus temperature were approximately −0.028 cm^−1^/°C and −0.06 cm^−1^/°C, respectively. This trend was similar for the multi-layer samples. In addition to the peak shift, a decrease in the peak intensity in the 2D band was observed. Figure 2d shows the 2D/G ratios. We observed that the intensity ratio weakened as temperature increased. An increasing background of the Raman spectra was also observed during the temperature increase. Various reasons are possible, this background would come from the heater and hot emission. Thus, the Raman measurements were taken after measuring the baseline at each temperature. The full width at half maximum (FWHM) of the G peak was 22 cm^−1^ at RT, but as the temperature increased, the FWHM also increased to about 30 cm^−1^. Considering that the number of phonons occupied is expressed by the Bose–Einstein distribution equation, the number of phonons occupied increases with increasing temperature. At higher temperatures, higher energy (higher frequency) phonons are also excited, resulting in an increase in phonon interactions. Furthermore, the increase in phonon population is thought to affect the peak position, intensity, and width (FWHM) of the Raman scattering, as observed in the present measurement. Therefore, it can be interpreted that the graphene was thermally and chemically stable in the observed range without any influence from the SiO_2_ substrate, change in sp_2_ bonds, or damage to the graphene. Based on these results, we confirmed that the peak shift only occurs upon heating, regardless of the number of layers. On the other hand, a slight temperature-dependent peak shift and a decrease in peak intensity at higher temperatures were observed in the D-band observed in the monolayer graphene in Figure 2a. This is thought to be due to the different generation processes of the G-band peak, which originates from the in-plane motion of carbon atoms, and the D-band peak, which originates from lattice motion away from the center of the Brillouin zone [13,14,15]. The D-band peak shift is caused by the lengthening of carbon–carbon bonds due to thermal expansion, which results in a decrease in the energy of lattice vibrations (phonon mode) and thus a peak shift to the lower wavenumber side. Alternatively, defect repair or changes in defect type and distribution may affect the phonon vibrational energy, resulting in a shift to the low wavenumber side. However, after cooling down, the peak was observed again at the same position as before heating, which is considered to be an effect of thermal expansion. The optical images of graphene and multilayer graphene on SiO_2_ substrate did not change significantly before and after heating. Referring to the relationship [16] regarding the number of layers and optical contrast, it is assumed that this is because the reflectivity of graphene itself does not change when heated.

### 3.2. Raman Spectra of Graphene on Cu Foil

**Figure 3:**
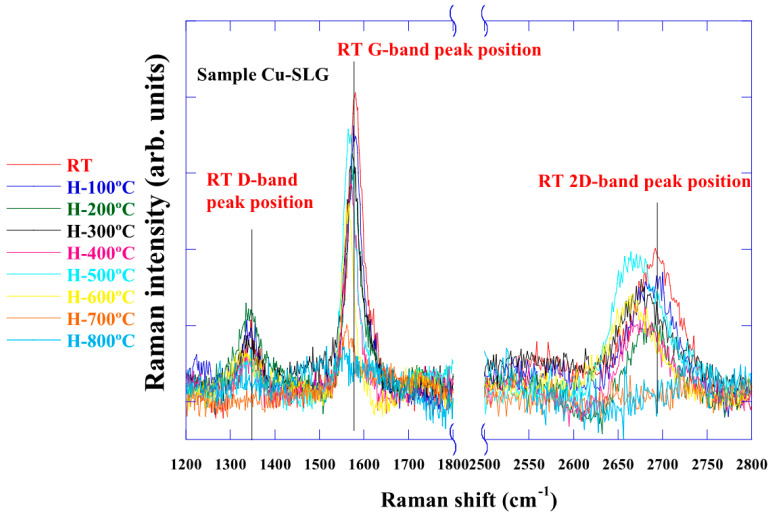
In situ Raman spectra of graphene on Cu foil. The peaks shifted up to 700 °C, similar to those on SiO_2_. However, Raman spectra were not observed, even during cooling.

**Figure 4:**
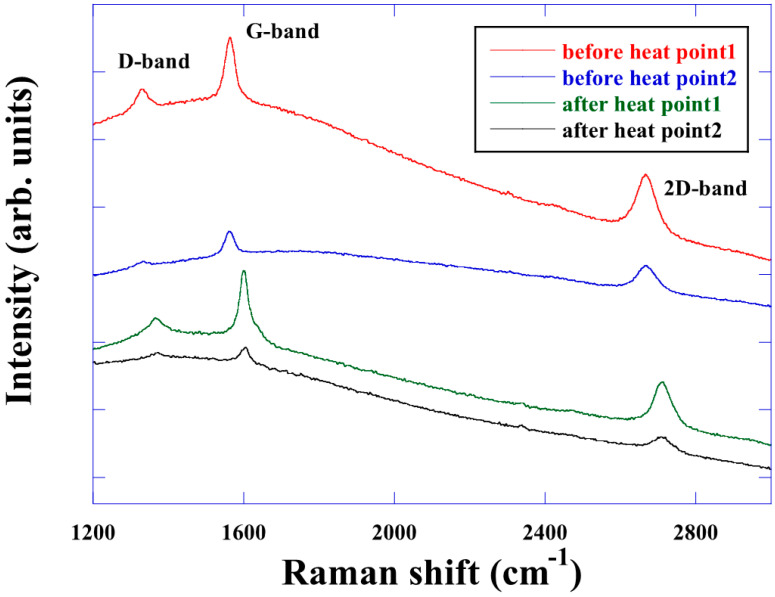
Raman spectrum after exposure to air. Raman spectra were not observed after 700 °C, but spectra were obtained when the sample was removed from the chamber.

Figure 3 shows the Raman spectra of graphene heated from RT to 900 °C. Up to 700 °C, a peak shift was observed, similar to that of graphene on SiO_2_. However, the G-band disappeared above 800 °C. Furthermore, Raman spectra could not be observed at 900 °C nor after cooling. Annealing the sample (700–800 °C) has been reported to remove the Cu oxide and restore the crystallographic features of copper surrounding the intact graphene [17], which may have led to the loss of the spectra. The sample was exposed to air, and its Raman spectrum was measured again, as shown in Figure 4. Studies report that, when graphene is exposed to air after vacuum heat treatment, hole concentration increases significantly [18]. Similarly, we observed the reappearance of the G-band and 2D band. From this result, the following is suggested at higher than 700 °C.

First, the measured graphene sample was either damaged or dissolved into Cu, where carbon has low solid solubility. Second, the Raman spectrum was no longer observed due to the change in the state of graphene surface or in the graphene–Cu foil interface caused by heating. Our previous studies’ optical microscopy images also show the surface in the same temperature range [19]. We believe that the Raman spectrum is no longer observed due to factors such as the desorption of intercalated oxygen, crystal growth of Cu domains, and strain relaxation.

### 3.3. Raman Spectra of Graphene on Ni Foil

**Figure 5:**
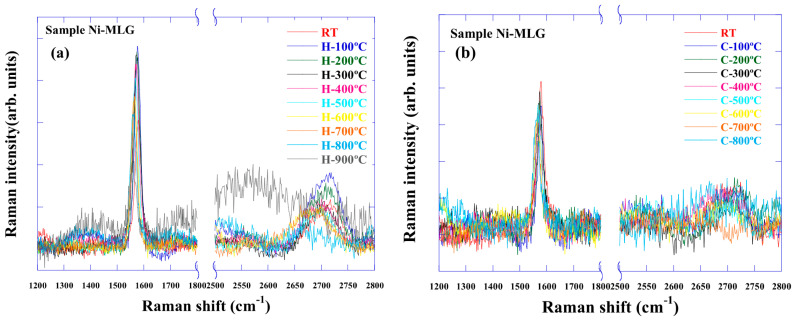
In situ Raman spectra of graphene on Ni foil. (**a**) shows the Raman spectrum upon heating, and (**b**) shows the Raman spectrum upon cooling.

Figure 5 shows the Raman spectrum of graphene on Ni foil. The peak shift with increasing temperature is similar to that observed for graphene on SiO_2_. In contrast to graphene on Cu, the G-band signal remains visible up to 900 °C, while the intensity of the 2D band decreases with increasing temperature. This decreases in 2D band intensity may be due to the dissolution of graphene layers into Ni, catalyzed by the Ni foil, effectively reducing the number of layers. Previous studies indicate that graphene on Ni begins to decompose at around 750 °C [20]. Moreover, our previous studies’ optical microscopy images also show surface in the same temperature range [19]. Comparing the Raman spectra during heating and cooling, it is thought that the decomposition and dissolution into metal during heating and the re-deposition during cooling changes the number of layers, which in turn changes the shape of the spectrum.

## 4. Conclusions

Graphene on insulating substrates remained stable with temperature changes, confirming its thermal and chemical stability. The peak shifts observed in the Raman spectra suggest that thermal expansion of the metal substrates influences the behavior of graphene during heating, or that graphene itself undergoes thermal expansion. For graphene on substrates with high catalytic activity and carbon solubility, significant changes in the Raman spectrum were observed. This behavior is likely due to the partial dissolution of graphene into the metal upon heating, followed by re-growth through re-deposition during cooling. These findings, in addition to enabling real-time observation of graphene growth, will contribute to the optimization of the graphene growth process with a simple optical system. In the future, the developments in image processing and AI integration could lead to more accurate and automated graphene identification.

## Data Availability

The original contributions presented in this study are included in the article. Further inquiries can be directed to the corresponding author.
